# Improving the accuracy of malaria-related laboratory tests in Ghana

**DOI:** 10.1186/1475-2875-3-38

**Published:** 2004-11-01

**Authors:** Imelda Bates, Veronica Bekoe, Alex Asamoa-Adu

**Affiliations:** 1 Liverpool School of Tropical Medicine, Pembroke Place, Liverpool L3 5QA, UK; 2 Public Health and Reference Laboratory, Korle Bu Teaching Hospital, Accra Central, PO Box 4456, Ghana; 3 Ghana Laboratory Service, Ministry of Health, Accra Central, PO Box 4456, Ghana

## Abstract

**Background:**

Inaccurate malaria results can lead to patient mismanagement, misperceptions about malaria resistance patterns and public health misinformation. All laboratories need to be able to demonstrate that their results are accurate. Establishing and maintaining a system for monitoring test accuracy is a complex, expensive and technically demanding process, which very few poor countries have been able to implement. This study described the process and assessed the feasibility of establishing a nation-wide system for improving the accuracy of malaria-related tests in peripheral laboratories in Ghana.

**Programme implementation:**

A baseline survey of all 693 laboratory staff in 205 sub-regional government and mission health laboratories in Ghana was conducted by a national network of laboratory supervisors. Survey results guided a training programme to improve test accuracy. Outcomes included changes in the quality of laboratory tests and the system was considered to be feasible if >50% of laboratory staff in each region received training and if test accuracy could be documented.

**Programme indicators:**

74% (mean) of the 693 laboratory staff were assistants with no professional qualifications. There were marked differences between regions in the availability of essential resources for malaria diagnosis (e.g. microscopes). 93% of laboratory staff received training; in six months there were increases of 11% and 7% respectively in the number of laboratories producing haemoglobin and malaria microscopy results of acceptable quality.

**Conclusions:**

It is possible to establish a system for improving and monitoring test accuracy in peripheral laboratories on a country-wide basis in a developing country using a model that could be adapted for use in other countries and for other components of health care provision.

## Background

Clinical laboratory services are a critical component of health systems. They are essential for patient management and for providing accurate public health data including early detection of malaria resistance. In many poorer countries, laboratory services have been neglected due to chronic under-investment. The emergence of drug-resistant malaria and the extra burden of supporting diagnosis and treatment of HIV/AIDS has increased the strain on laboratories. The widespread resistance of malaria to cheap, antimalarial drugs such as chloroquine and the increasing use of relatively expensive combination therapy, means that presumptive treatment of all fevers as malaria may no longer be a sustainable option for some countries [1]. It is, therefore, imperative that malaria diagnosis at peripheral level health facilities, where the greatest burden of malaria health care provision is focused, is accurate.

Establishing and maintaining an accurate and reliable laboratory service is a complex, expensive and technically demanding process, which very few poor countries have been able to implement. It depends on good laboratory management to oversee processes such as documentation, audit cycles, quality assurance and external validation, safety practices, and supervisory and accountability structures [2] and should be combined with improvements in clinical practice. Sub-standard laboratory services waste public and individuals' resources and result in clinical mismanagement and inaccurate health information. They also generate a culture of mistrust and communication breakdown between laboratory and clinical staff which contributes to low morale within the technical profession. Poor quality laboratory services have the greatest impact on the poorest people who use the service because they have the largest burden of ill-health [3]. To our knowledge there is no comprehensive nationwide system for monitoring the accuracy of malaria-related laboratory tests (i.e. malaria microscopy, haemoglobin estimation, tests associated with blood transfusion) currently operational in any sub-Saharan country.

In January 2000, the Ministry of Health in Ghana commenced a two-year programme to determine the feasibility of establishing a nationwide quality assurance system for common tests performed at peripheral (district and sub-district) laboratories. This programme was specifically designed to complement the Ministry's 5-year Programme of Work [4]. In Ghana, laboratory services exist at most levels of health facility, except smaller health centres. Ghana's decentralization policy means that regional and district health managers are responsible for delivering and evaluating health care provision, including laboratory services. Prior to this programme there were no local or national quality assurance systems and no functional supervisory network for laboratories in Ghana. This feasibility programme was built on established laboratory management structures and locally available resources and was implemented by a national network of senior laboratory technicians. The aim of the programme was to determine the feasibility of establishing a nationwide system for improving the accuracy of malaria and other common laboratory tests. All staff performing laboratory tests in all public sector peripheral laboratories in Ghana were included in the programme irrespective of their grade.

## Programme implementation

### Establishing a national network of laboratory 'supervisors'

Two senior technicians from each of Ghana's ten administrative regions were chosen by the Ministry of Health to constitute the national network of laboratory supervisors who would implement the programme. They were selected on the basis of their geographic location, seniority and commitment to improving laboratory services. All the supervisors had technical qualifications (2–3 years certificate or diploma course), but none had any higher technical or educational qualifications.

### Programme objective and baseline survey

A workshop for the supervisors was held in Ghana at the beginning of the programme to define the objectives and to develop a workplan, timetable and monitoring processes. The agreed programme objective was to establish a system in all peripheral government laboratories in Ghana, for monitoring the accuracy of results of malaria microscopy, haemoglobin estimation and other commonly performed tests. The ability to train over 50% of laboratory staff in each of the ten regions and to monitor changes in the accuracy of results, were used as indicators of programme feasibility. As very little information was available about the state of Ghana's laboratories, supervisors initially carried out a nationwide baseline survey of all Ghana's peripheral laboratory facilities. They personally visited every government and mission laboratory in Ghana and collected first hand information about staff, equipment and tests offered. For each laboratory they also completed a safety checklist based on the World Health Organization's recommendations for laboratory safety [5].

### Programme design and methods

#### Programme planning and training

Ideally training should be targeted towards the tests which are performed most poorly. However, as there were no monitoring systems in place in Ghana for laboratory tests and none of Ghana's laboratory staff had had any experience of these systems, it was not possible initially to identify the worst performed tests. The supervisors therefore started by providing training on seven of the most commonly performed tests. They used Ghana's Standard Laboratory Operating Procedures [6], which describe nationally standardized methods for individual tests, as their core teaching manual. Their choice of teaching methods, predominantly combinations of workshops and workplace training, varied between regions depending on local resources, needs and geography.

#### Monitoring test accuracy

Quality assurance systems used in industrialised countries and published in the literature are not appropriate for rural laboratories in poor countries such as Ghana. They are too complex for a workforce that is primarily made up of laboratory assistants and they are based on assumptions that the methods are generally automated and communication and transport networks are reliable. The supervisors therefore had to devise workable methods for externally monitoring test results from the peripheral laboratories. They distributed samples with known values to peripheral laboratories who processed them under normal working conditions. Once the methods for distributing, preserving and measuring test accuracy had been optimized for local use they were piloted in a small number of laboratories, before being introduced throughout each region. The ways in which the test accuracy was determined varied for each test. For example, for haemoglobin estimations a whole blood sample with known value (determined by repeated measurements in a regional laboratory) was distributed to peripheral laboratories and supervisors decided that laboratories with results outside the target value ±10% would receive priority for training. As the quality of results from peripheral laboratories improved, the supervisors reduced the acceptable range of results to ±5% of the target value. For malaria microscopy, the target malaria result of a whole blood sample or malaria smear was determined by consensus of several technicians from the regional hospital. Results from peripheral laboratories were considered 'accurate' if laboratories reported the presence (or absence) of malaria parasites and quantified them to within one grade (on a grading system of 0,+,++,+++) of the target result. For sickle cell tests a blood sample of know sickle status, determined by the regional laboratory, was distributed. Accurate results were those that correctly identified the sample as containing sickle haemoglobin or not. If the majority of results from peripheral laboratories for a single test varied from the target result, the supervisors re-evaluated their test target values. Whole blood samples were distributed in order to check the complete process of testing (e.g. pipetting accuracy, malaria smear preparation) rather than just an individual component. Supervisors used results of the quality monitoring to provide constructive feedback to the laboratory staff and to target their training towards tests and laboratories that performed particularly poorly.

#### Programme funding

Initially the programme was funded directly from Ministry of Health headquarters but subsequently, through collaboration with regional Ministry of Health administrators, several supervisors were able to access their own regional funds.

### Programme indicators

#### Baseline survey

There were 205 laboratories in Ghana located in regional and district government hospitals (97), mission hospitals (53) or health centres (54). The total staff complement in these laboratories was 693. The percentage of staff in each of the ten regions with no professional qualifications (assistants or bench-trained with 6 weeks to 1 year training) varied from 60–83% (mean 74%). Even after correcting for differences in regional populations and excluding regions with teaching hospitals, there were wide variations between regions in the number of laboratories and the availability of essential laboratory equipment such as microscopes, (Table 1). The safety survey showed that over 50% of 62 laboratories surveyed lacked essential items such as automatic pipettes (necessitating mouth-pipetting) (74%), protocols for waste disposal and equipment maintenance (82% and 100% respectively), first aid kits (100%) and fire safety equipment (94%). Other unsafe practices included allowing patients inside the laboratory (89%) and eating and drinking within the laboratory (55%).

**Table 1:** Regional variations in laboratory resources corrected for population (year 2000) (excluding two regions with teaching hospitals)

**Region**		**2**	**3**	**4**	**6**	**7**	**8**	**9**	**10**	**Mean**	**Range**
**Population (millions)**		2.10	1.55	2.65	2.10	1.25	0.70	1.80	1.85		
**Laboratories**	Total	16	15	40	7	26	9	57	32		
	per 100,000 pop.	0.76	0.97	1.51	0.33	2.08	1.29	3.17	1.73	**1.48**	**0.33–3.17**
**Trained staff**	Total	13	9	21	11	11	7	19	24		
	per 100,000 pop.	0.62	0.58	0.79	0.52	0.88	1.00	1.06	1.30	**0.85**	**0.52–1.30**
**Microscopes**	Total	30	20	51	NI	16	6	76	20		
	per 100,000 pop.	1.40	1.29	1.92	NI	1.28	0.86	4.22	1.08	**1.72**	**0.86–4.22**
**Colorimeters**	Total	17	8	46	6	6	5	14	14		
	per 100,000 pop.	0.81	0.52	1.74	0.29	0.48	0.71	0.78	0.76	**1.22**	**0.52–1.74**
**Centrifuges**	Total	20	15	46	12	6	6	54	21		
	per 100,000 pop.	0.95	7.42	1.74	0.57	0.48	0.86	3.00	1.14	**2.02**	**0.48–7.42**
**Haematocrit centrifuge**	Total	9	4	39	7	8	2	5	10		
	per 100,000 pop.	0.43	0.23	1.47	0.33	0.64	0.29	0.28	0.54	**0.53**	**0.23–1.47**
**Spectrophotometer**	Total	5	3	13	2	5	2	3	2		
	per 100,000 pop.	0.24	0.19	0.49	0.95	0.40	0.29	0.17	0.11	**0.39**	**0.11–0.95**
**Blood mixer**	Total	5	3	20	0	0	1	10	0		
	per 100,000 pop.	0.24	0.19	0.75	0	0	0.14	0.56	0	**0.24**	**0–0.75**

NI = no information

#### Extent of training and impact on test accuracy

After 18 months, a mean of 93% (regional variation 58%–100%) of all Ghana's laboratory staff had been trained in malaria-related and other common tests. During the final six months of the programme the supervisors monitored the quality of haemoglobin estimations, sickle cell test and malaria microscopy results in 48% of all Ghana's 205 laboratories. In 4 regions the quality of up to 11 tests had been monitored. Tests that gave quantitative results were consistently the most poorly performed tests in all regions with only 78%, 78% and 84% of laboratories producing acceptable results for haemoglobin measurements, white blood counts and malaria microscopy respectively. Tests for HIV, hepatitis B, sickle-cell screen and ZN stain were consistently performed well with over 95% of laboratories meeting agreed target results. After a further six months training there were improvements in the accuracy of several tests, particularly in haemoglobin estimation and malaria microscopy with 89%, 83% and 91% of laboratories producing acceptable results for these tests respectively, (Table 2).

**Table 2:** Changes in test accuracy in six months

	**Mean number of laboratories with accurate results/total surveyed (%)**	
**Test**	**Month 1**	**Month 6**

Haemoglobin	45/58 (78)	49/58 (89)
Malaria microscopy	49/58 (84)	49/54 (91)
Sickle screen	53/53 (100)	51/51 (100)
White blood count	31/40 (78)	40/48 (83)
Blood group	23/24 (96)	57/58 (98)
Cross match	7/7 (100)	7/7 (100)
HIV test	27/28 (96)	28/28 (100)
Hepatitis B antigen	22/22 (100)	40/40 (100)
ZN stain	38/39 (97)	39/39 (100)

## Discussion

There is very little information available about the state and quality of laboratory services in peripheral health facilities in poorer countries. The baseline survey showed that in Ghana three quarters of public sector laboratory staff do not have any technical qualifications, and even the supervisory cadre only have Diplomas. Through this study we have shown that the lack of professional qualifications amongst the laboratory workforce and the inequitable distribution of laboratory equipment, are not obstacles to establishing a simple and far-reaching quality assurance process.

This study has shown that the worst performed tests at sub-regional level are those that generate quantitative or subjective results (such as haemoglobin estimation and malaria microscopy) rather than simple 'positive' or 'negative' results (such as HIV and hepatitis B screening tests). Further research is needed to examine the cost-effectiveness of potentially more accurate or less complex tests (e.g. malaria rapid diagnostic tests, HemoCue). Although these tests may be more expensive than those in use in most districts, their simplicity and accuracy may save downstream costs. One of the major limitations of this study is the lack of quality assurance at regional and supra-regional level for common laboratory tests. These were the laboratories responsible for determining the target values of tests distributed to peripheral laboratories. To overcome this problem, regional laboratories tested the samples several times before determining the target values and they also exchanged samples with each other as an inter-regional check on results. Plans are in place to expand this into an inclusive national quality monitoring system for higher level laboratories.

We have confirmed previous work showing that at peripheral health facilities haemoglobin using manual methods is the most inaccurate test [7]. Safety issues are often not a priority for laboratories in poorer countries but overcrowding and poor laboratory organisation are recognized to be associated with a significant risk of accidents and consequent infection of health workers. Many of the safety issues identified through this project could be easily and cheaply rectified but because there was no supervisory system in place they had been ignored.

To our knowledge this is the first external laboratory quality assurance programme in sub-Saharan Africa to demonstrate that it is feasible to achieve widespread coverage of peripheral health laboratories and staff. Through this programme an effective and practical model has been developed for improving the accuracy of common laboratory tests in Ghana. The model is based on a national network of senior technicians who implemented and supervised a continuous cycle of test monitoring and targeted training for all laboratory staff in their regions. The programme's success was dependent on the quality and commitment of the national network of laboratory supervisors. Participation in the programme was itself a strong motivational force for the supervisors. Because of the supportive attitude of the supervisors, the laboratory staff viewed the monitoring visits as educational rather than punitive and this ensured cooperation when remedial action was required. Health planners can use the lessons learnt from this programme to introduce measures to improve morale and job satisfaction of key health workers especially those in the neglected laboratory services. Components of this model have already been adopted by other countries for their laboratory programmes and could be adapted for other health disciplines in poorer countries.

Several steps are necessary before this model could be sustainably implemented. The network of supervisors needs to have pro-active long-term support from all stakeholders, combined with career and promotion packages. Health managers need to provide secure funding for laboratory quality assurance programmes at all levels. This includes establishing internal quality control measures and external validation systems that are linked into national and international external quality monitoring schemes. To be successful, this model will require prioritization of the laboratory service by policy makers at national and supra-national level and adequate representation of the laboratories in decision-making processes. Implementing a quality assurance system for laboratories in poorer countries is expensive and logistically complicated. Managers will need to balance cost against quality, taking into account that it is those who are most vulnerable to ill health who can least afford to bear the brunt of the consequences of inaccurate laboratory results.

## Authors' contributions

IB and VB designed the outline framework for the programme. IB documented and collated results. VB was the local programme coordinator. AAA provided an overview and ensured the programme was compatible with Ministry of Health policies. All authors read and approved the final manuscript.

## Funding sources

This study was supported by the Ministry of Health in Ghana and the Malaria Knowledge Programme (funded by the Department for International Development, UK) at the Liverpool School of Tropical Medicine. The Department for International Development had no role in study design, the collection, analysis and interpretation of data, in the writing of the report or in the decision to submit the paper for publication and accepts no responsibility for the information or views expressed.

## References

[B1] Barnish G, Bates I, Iboro J (2004). Newer drug combinations for malaria. BMJ.

[B2] World Health Organization (1995). Quality Systems for Medical Laboratories.

[B3] Ghana health statistics (1998).

[B4] Ministry of Health, Government of Ghana (1996). Health Sector Programme of Work 1997–2001.

[B5] World Health Organization (1997). Safety in health care laboratories.

[B6] Public Health and Reference Laboratory Ministry of Health, Ghana (1998). Standard Laboratory Operating Procedures.

[B7] Mundy C, Bates I, Nkhoma W, Floyd K, Kadewele G, Ngwira M, Khuwi A, Squire SB, Gilks CF (2003). The operation, quality and costs of a district hospital laboratory service in Malawi. Trans R Soc Trop Med Hyg.

